# Tau Oligomers as Potential Targets for Alzheimer’s Diagnosis and Novel Drugs

**DOI:** 10.3389/fneur.2013.00167

**Published:** 2013-10-28

**Authors:** Leonardo Guzmán-Martinez, Gonzalo A. Farías, Ricardo Benjamin Maccioni

**Affiliations:** ^1^Laboratory of Cellular and Molecular Neurosciences, Faculty of Sciences, University of Chile, Santiago, Chile; ^2^International Center for Biomedicine (ICC), Santiago, Chile; ^3^Department of Neurology and Neurosurgery North, University of Chile, Santiago, Chile; ^4^Department of Neurological Sciences East, Faculty of Medicine, University of Chile, Santiago, Chile

**Keywords:** tau oligomers, PHFs, Alzheimer’s disease, tauopathies, diagnosis and treatment

## Abstract

A cumulative number of approaches have been carried out to elucidate the pathogenesis of Alzheimer’s disease (AD). Tangles formation has been identified as a major event involved in the neurodegenerative process, due to the conversion of either soluble peptides or oligomers into insoluble filaments. Most of recent studies share in common the observation that formation of tau oligomers and the subsequent pathological filaments arrays is a critical step in AD etiopathogenesis. Oligomeric tau species appear to be toxic for neuronal cells, and therefore appear as an appropriate target for the design of molecules that may control morphological and functional alterations leading to cognitive impairment. Thus, current therapeutic strategies are aimed at three major types of molecules: (1) inhibitors of protein kinases and phosphatases that modify tau and that may control neuronal degeneration, (2) methylene blue, and (3) natural phytocomplexes and polyphenolics compounds able to either inhibit the formation of tau filaments or disaggregate them. Only a few polyphenolic molecules have emerged to prevent tau aggregation. In this context, fulvic acid (FA), a humic substance, has potential protective activity cognitive impairment. In fact, formation of paired helical filaments *in vitro*, is inhibited by FA affecting the length of fibrils and their morphology.

## Introduction

Tau protein, a member of the microtubule-associated protein (MAPs) family, plays a fundamental role in the assembly and stabilization of microtubules, as well as on axonal transport and neurite outgrowth (1–3). In this context, tau protein plays an important role in the maintenance of neuronal polarity and in the stabilization of the morphology of differentiated neurons. In developing neurons, tau activity is crucial for the morphogenesis of the growth cones and, it has been suggested to play a key role in promoting axonal growth (4). Tau is encoded by a single gene located on chromosome 17 (17q21), possessing 16 exons in its primary transcript. Six different isoforms are expressed by post transcriptional modifications (alternative splicing) from the primary transcript. Mature protein length is about 352 up to 441 amino acid residues, and a molecular weights of 45–65 kDa depending on the tau isoforms (5, 6). The C-terminal region has a domain containing the microtubule-binding repeats, which is critical for microtubule assembly (2), whereas the affinity of tau for microtubules is finely regulated by an orchestrated set of phosphorylations. The motif KXGS is one of several different motifs located within these repeats susceptible to be phosphorylated (7). In turn, tau is characterized as an hydrophilic cationic protein, unfolded under native conditions, and with a low ordered secondary structure (8). In addition to roles in stabilization of microtubules, tau plays a major role in bridging the different cytoskeletal structures. Thus, besides microtubules, tau interacts with actin and intermediate filaments (2, 9).

Tau is located primarily in neurons, however, traces of this protein have been found in certain non-neuronal cells. Under pathological conditions, tau can be also expressed in glial cells (10). It is also possible to find tau or it’s mRNA in several peripheral tissues such as heart, liver, lung, skeletal muscle, among others (11–13). Interestingly, tau variants have been also observed in human platelets (3, 14, 15).

Tau phosphorylation plays an important role in regulating its binding to microtubules and thereby regulating their stability within neuronal cells. However, under pathological situations, tau protein is abnormally phosphorylated or dephosphorylated in specific residues, perhaps due to the activities of various protein kinases and phosphatases (such as GSK-3β and Cdk-5, and PP1A and PP2, respectively) (16, 17). This change in the phosphorylation state of tau can lead to irreversible changes in the dynamics of microtubules, cellular dysfunction, ultimately triggering cell death of the neuron (6, 18). Moreover, tau protein may have other post-translational modifications, including: (i) glycosylations, (ii) ubiquitinations, (iii) truncations, and (iv) nitrations (6).

Hyperphosphorylated tau protein is the main component of abnormal protein aggregates found in the neurons of patients with neurodegenerative brain disorders known as tauopathies. Those neurodegenerative diseases have in common the presence of intraneuronal aggregates of tau. These aggregates are known as Neurofibrillary Tangles (NFTs) and are made up of paired helical filaments (PHFs) and straight filaments mainly composed of hyperphosphorylated tau. The formation of PHFs from tau molecules may follow different steps and could involve tau phosphorylations, followed by limited proteolysis and conformational changes in tau protein (19), and finally its polymerization into aberrant polymers in neuronal cells. These neuronal disorders include: Pick’s disease, corticobasal degeneration (CBD), progressive supranuclear palsy (PSP), frontotemporal dementia with Parkinsonism linked to chromosome 17 (DFTP-17) and Alzheimer’s disease (AD). AD is considered the most prevalent tauopathy worldwide (20). In AD, abnormal phosphorylations occur and specifically tag certain amino acids of tau protein: Ser202, Thr205, Ser235, and Ser404 (21). These post-translational modifications are catalyzed by two main protein kinases: the Cdk-5/p35 system and GSK-3β (6). In this paper, we focus on how tau oligomers became the focus for the search for new drugs, and also potential targets for accurate diagnosis of AD.

## Are Tau Oligomers Responsible for Neuronal Degeneration? Technical Approaches

Insoluble aggregates of the MAP tau characterize a number of tauopathies. Although there is much evidence linking tau to neurodegeneration, the precise mechanism of tau-mediated neurotoxicity remains to be elucidated. In fact, tau oligomers appear to be the toxic form of tau in neurodegenerative disease. In agreement with this hypothesis, the presence of immunoreactive tau protein in neurons of AD brain tissue, previous to tangle formation, has been shown. Binder and coworkers have produced the novel monoclonal antibody TOC1 that recognizes non-fibrillary tau. This antibody is selective in terms of specifically labeling tau dimers and oligomers, but does not label tau filaments. Time-course analysis and antibody labeling indicates that oligomers appear as an early event in AD pathogenesis. Aggregated tau, but not monomeric tau, inhibited anterograde fast axonal transport. This inhibition requires a small stretch containing amino acids from the N-terminal region on tau, a phosphatase-activation domain. The molecular chaperone heat-shock protein 70 (Hsp 70) clearly affects tau oligomers formation and stability, as investigated in the squid axoplasm. Hsp 70 preferentially bound to tau oligomers over filaments and prevented anterograde axonal transport inhibition observed with a mixture of both forms of aggregated tau (22).

Tau aggregates comprise abnormally hyperphosphorylated and misfolded tau. Research has traditionally focused on understanding how hyperphosphorylated and aggregated tau mediates dysfunction and toxicity in tauopathies. Recent findings in *Drosophila* and rodent models of tauopathy suggest that large insoluble aggregates such as tau filaments and tangles may not be the key toxic species in these diseases (23). Thus, some investigators have shifted their focus to study pre-filament tau species such as tau oligomers and hyperphosphorylated tau monomers. Interestingly, tau oligomers can exist in a variety of states including hyperphosphorylated and unphosphorylated forms, which can be both soluble and insoluble. It remains to be determined which of these oligomeric states of tau are causally involved in neurodegeneration and which molecule signal the beginning of the formation of inert/protective filaments. It will be important to better understand this aspect so that tau-based therapeutic interventions can target the really toxic tau species.

Another interesting study showed that oligomers of recombinant full-length human tau protein are neurotoxic *in vivo* after subcortical stereotaxic injection into mice. Data showed that tau oligomers impaired memory consolidation, whereas tau fibrils and monomers did not. In this context it was assumed that tau oligomers can affects synaptic transmission. Thus, synaptic dysfunction seems to result from the action of tau oligomers, potentially reducing the activity of the synaptic vesicle-associated proteins synaptophysin (24). Some studies identify tau oligomers as an acutely toxic tau species *in vivo*, and suggest that tau oligomers induce neurodegeneration by affecting mitochondrial and synaptic function, both of which are early hallmarks in AD and other tauopathies (25). These results open new avenues for neuroprotective intervention strategies for tauopathies by targeting tau oligomers.

## Oligomers and Their Usefulness for Monitoring AD

**Figure 1:**
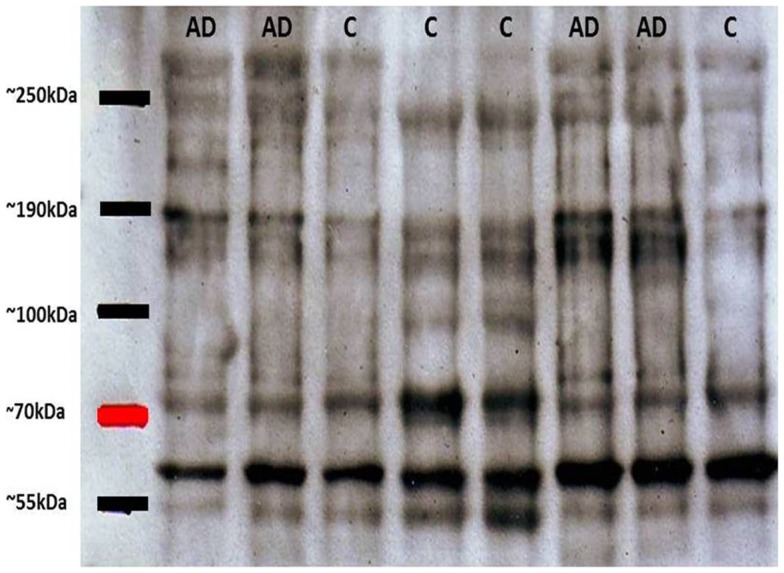
**Representative immunoblots of platelet tau with tau-5 antibody**. High molecular weight tau bands (about 80 kDa) can be appreciated, with greater immunoreactivity in patients with Alzheimer’s disease (AD) compared with control subjects (C). Subsequent densitometric analysis allows obtaining the relationship between HMWtau versus LMWtau.

As mentioned above, tau oligomers are one of the neuropathological hallmarks of AD and other tauopathies. Regarding NFT accumulation in AD, there is evidence showing that progressive neuronal loss and cognitive impairment correlates with the accumulation of soluble species of tau in NFTs in AD mouse models (26). This etiopathological feature has become the target for potential treatments for AD, diagnosis and monitoring evolution of the disease. On this last point, some diagnostic biomarkers for AD based on tau have been developed: (1) biomarkers in cerebrospinal fluid (CSF) and (2) biomarkers in platelet tau (3). Since in the CSF significant exchange of substances with a varied neural environment appears to occur, tau variants are released to the fluid. In this context, one of the most reliable biomarkers reported was based on the ratio between normal tau and hyperphosphorylated tau (P-tau) in the CSF (27). This evaluation about tau and P-tau allows a better correlation with the later stages of synaptic dysfunction and early neurodegeneration (27, 28). Furthermore, the second biomarker is based on the detection of tau in blood platelets (15, 29). Alterations in platelets from AD patients, including modifications in platelets β-amyloid Precursor Protein (APP) have been described previously (30) and APP evaluations have been postulated as a potential biomarker for AD (31). Considering that information we focused our efforts in analyses of platelets tau protein. The innovative method was developed in our laboratory and is based upon the difference between the ratio of molecular species of high molecular weight tau (multimeric) versus low molecular weight (monomeric). In western blot experiments performed with platelet protein extracts obtained from peripheral blood from healthy subjects and patients with AD, it was observed that the latter had tau immunoreactive bands migrating at molecular weights much higher than expected by electrophoresis under denaturing and reducing conditions (SDS-PAGE) (15). These high molecular weight forms of tau (tau HMW) could be attributed to oligomeric forms of the protein, which are increased in AD patients as compared to healthy elderly subjects (Figure 1). Therefore, platelets tau has been postulated as a biomarker for AD (29). Different tau species with variable stages of aggregation are visualized in the electrophoretic patterns. Subsequent studies by Farias et al. (15), have shown that there is a close correlation between the degree of platelet tau modification and level of cognitive impairment, which was measured using neuropsychological tests in patients with AD [for more detailed information about these biomarkers see Ref. (3)]. Moreover, correlations between HMWtau/LMWtau ratio and brain neuroimaging have been observed. This ratio, controlled for age and education, significantly correlated with a clusters of 717 voxels in the right parahippocampal cortex with peak at Talairach coordinates 16, −10, −23 (unpublished observations).

## Tau as a Therapeutic Target for Anti-Alzheimer Drugs

Since tau has been recognized as an important actor in neurodegenerative diseases, many molecules that act on tau pathology have been investigated as potential vectors for therapeutic approaches for AD, but also for other tauopathies such as PSP or CBD. One of the molecules that have attracted much attention is the phenothiazine methylthioninium chloride, better known as methylene blue (MB). MB has a very interesting property as an aggregation inhibitor for proteins that adopt beta sheet conformation (32).

**Figure 2:**
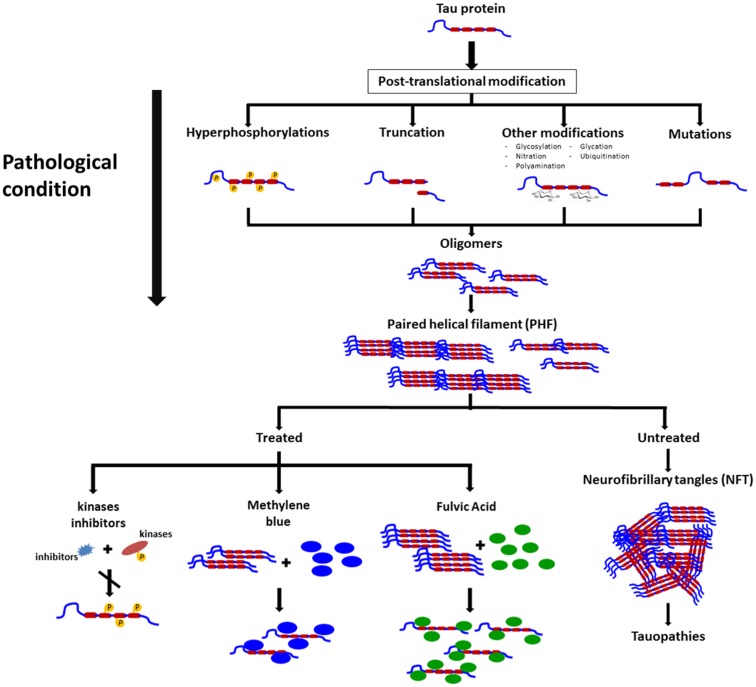
**Schematic representation on the changes in tau leading to pathological conditions, formation of neurofibrillary tangles, and some molecules that exert their actions on polymeric forms of pathological tau and that appear to prevent polymers formation and possible disassembly of these filaments**.

Rember™ is the trade name of MB, and a Phase II study randomizing 321 mild to moderate AD patients with placebo or three different doses of Rember™ was presented in 2008 at the International Conference on AD in Chicago. Treatment effects of four points were described on ADAS-Cog on the treatment group as compared to placebo subjects, and also conservation on the cerebral blood flow and brain glucose used in SPECT and FDG-PET scans (33). However, as today, the lack of peer reviewed publications on this compound affect the reliability of the results. In animal models, the effects of MB on cognition appears not to be related to a reduction on NFTs but to a decrease in soluble tau levels (34) (Figure 2).

Other groups have focused their efforts on investigating tau kinases inhibitors (16) or tau phosphatase activators (17), as an indirect manner to halt tau hyperphosphorylation, recognized as leading to pathological aggregation of the protein. However, difficulties in finding specific inhibitors/activators with adequate safety profiles have impacted in the lack of new drugs in this area (32, 35). A possible way to decrease adverse effects of tau kinase inhibition might be the use of relatively broad specificity, low power compounds (36).

Davunetide is an eight aminoacids peptide that can be administered intranasally or intravenously and has been described as a tau hyperphosphorylation inhibitor as well as an inhibitor of caspase 3 activation (37). Davunetide has been successfully evaluated in a series of *in vitro* and *in vivo* models (38) for neurodegenerative diseases that include AD, PSP, and schizophrenia (38, 39).

An example of “non-traditional” approaches to tau aggregation regulation includes chaperones modulation. Hsp 70 chaperones assist protein-folding processes and are found up-regulated in several tumors, but also in neurodegenerative diseases such as Pick’s disease, AD, and other tauopathies. Deregulation in Hsp 70 chaperones appears to be implicated in the processes of tau aggregation, so compounds that bind Hsp 70 chaperones are under investigation as possible treatment compounds for neurodegenerative diseases, since there is evidence that Hsp 70 inhibition leads to tau ubiquitination and clearance through ubiquitin-proteasome system (40).

There are some natural compounds that are able to inhibit tau aggregation and possibly, make an impact in neurodegenerative diseases. Shilajit is a natural phytocomplex that has been found in the Himalayan Mountains between India and Nepal and also in the Tibet and Afghanistan and has been used in ayurvedic medicine for centuries as a rejuvenating compound. Our laboratory has worked with the *Andean Compound* (or *Andean shilajit*), a natural compound that can be found in Andean Mountains. *Andean shilajit* is generated by a long-term degradation of certain plants by microorganisms, mostly fungi and is rich in Fulvic Acid (FA) and humic substances among others (Figure 2). This natural endemic phytocomplex, resulting from fossilized plants degradation through the years, was discovered in 2008 in the North of Chile and was named *Andean Compound* (41). *In vitro* assays and cell culture data show that Andean Compound and FA strongly interferes with tau aggregation, and interestingly an increase in neurites outgrowth has been observed in neural cell cultures exposed to this natural compound (42). In addition, a placebo-controlled pilot clinical study suggests that consumption of a nutraceutical formulation of *Andean Compound* plus B complex vitamins may produce stabilization of cognitive function in AD patients at a 24-weeks as determined with Global deterioration scale (GDS) and Neuropsychiatric inventory (NPI) measurements (42) (Figure 2). On the other hand the same *shilajit* – based compound has a very good safety profile when tested on healthy population (unpublished data).

## Concluding Remarks

Currently, we do not know the exact cause of synaptic dysfunction and neurodegeneration in AD, however, in recent years it has become increasingly clear the importance of tau protein and its post-translational modifications in the pathophysiological processes of AD and other tauopathies.

In this context, determination of different forms of tau protein in brain, CSF (43) and also in blood (44) and peripheral cells (15) has been postulated as a powerful tool for detection and monitoring of the disease in different stages and there is clear evidence of a profile of tau and other biomarkers modifications during AD progression (45, 46). The presence of tau modifications in peripheral cells also points to the inference that AD may be a systemic disease not only confined to nervous tissue. But currently we do not have any information on the functional impact – if any – of oligomeric tau forms in peripheral cells. The latest criteria of AD consider tau-based biomarkers as reliable indicators of neuronal injury processes (47), but as of today there is not a widely available marker of tau modification for clinical use. Hopefully in the near future we will refine techniques for non-invasive assessment of tau. Thereon, are extremely interesting attempts to generate PET markers for tau deposition in brain (48, 49) and we already have promising data regarding measurement of different forms of tau in blood cells (15, 29).

On the other hand, in the absence of positive clinical results in studies with beta amyloid targeted therapies, the need to evaluate therapies that can act on post-translational changes of tau protein has become evident. Particular interest has been paid to therapies that may modulate levels of phosphorylation and oligomerization of tau, whether by direct action on tau or by acting on other related proteins like kinases or heat-shock proteins.

Unfortunately we still need information from large multicenter studies about the usefulness of tau-focused therapies, however, this is a promising field that has attracted the efforts of multiple investigators, so we expect that new and exciting discoveries are right around the corner.

## Conflict of Interest Statement

The authors declare that the research was conducted in the absence of any commercial or financial relationships that could be construed as a potential conflict of interest.
